# Principles of Medical Jurisprudence: Designed for the Professions of Law and Medicine

**Published:** 1850-08

**Authors:** 


*Principles of Medical Jurisprudence:* designed for the professions of Law and Medicine. By Amos Dean, Counsellor at Law, and Professor of Medical Jurisprudence in the Albany Medical College. Albany: Gould, Banks & Gould, 475 Broadway. New York: Banks, Gould & Co., 344 Nassau st. 1850 8 vo. pp. 664.

The plan and scope of this work are succinctly set forth in the following extract from the preface:

An experience of some eleven years in teaching in this department, together with a knowledge of the wants of the legal and medical professions in regard to it, have led to the compilation of the work now offered to the public. It does not propose to add new heads, or general topics, for discussion ; to deal in original disquisitions, upon doubtful or unsettled principles ; or to offer mere novelties to those in pursuit of knowledge on its various subjects. The objects chiefly had in view, have been, a methodical, systematic arrangement of the topics legitimately embraced in the department ; and in the treatment of each, a condensation of the knowledge now possessed ; and an exhibition of it in a clear, natural, and logical order, together with such illustrations as were deemed necessary to make an application of the principles to practice. In the selection, and narration of cases, both multiplication, and minuteness of detail, have been equally avoided; the object being simply to illustrate, and facilitate the reduction to practice, of the principles embraced in them. By adopting this course, I am enabled, within the compass of a single volume, to state in concise terms, all, or nearly all, the ascertained facts and well settled principles that are important to be known, with a brief statement of the cases that serve to illustrate them ; making at the same time, a reference to the sources from which they were obtained, and where also a greater number of cases, and more minute details of each may be found.

The author's experience, and his acknowledged ability commend the work to the confidence of the medical and legal professions.

For sale by Derby & Co.

